# PAFAH1B1 haploinsufficiency disrupts GABA neurons and synaptic E/I balance in the dentate gyrus

**DOI:** 10.1038/s41598-017-08809-x

**Published:** 2017-08-15

**Authors:** Matthew T. Dinday, Kelly M. Girskis, Sunyoung Lee, Scott C. Baraban, Robert F. Hunt

**Affiliations:** 1 Epilepsy Research Laboratory, Department of Neurological Surgery, University of California San Francisco, San Francisco, USA; 2 Department of Anatomy & Neurobiology, University of California Irvine, California, USA

## Abstract

Hemizygous mutations in the human gene encoding platelet-activating factor acetylhydrolase IB subunit alpha (Pafah1b1), also called *Lissencephaly-1*, can cause classical lissencephaly, a severe malformation of cortical development. Children with this disorder suffer from deficits in neuronal migration, severe intellectual disability, intractable epilepsy and early death. While many of these features can be reproduced in Pafah1b1^+/−^ mice, the impact of Pafah1b1^+/−^ on the function of individual subpopulations of neurons and ultimately brain circuits is largely unknown. Here, we show tangential migration of young GABAergic interneurons into the developing hippocampus is slowed in Pafah1b1^+/−^ mice. Mutant mice had a decreased density of parvalbumin- and somatostatin-positive interneurons in dentate gyrus, but no change in density of calretinin interneurons. Whole-cell patch-clamp recordings revealed increased excitatory and decreased inhibitory synaptic inputs onto granule cells of Pafah1b1^+/−^ mice. Mutant animals developed spontaneous electrographic seizures, as well as long-term deficits in contextual memory. Our findings provide evidence of a dramatic shift in excitability in the dentate gyrus of Pafah1b1^+/−^ mice that may contribute to epilepsy or cognitive impairments associated with lissencephaly.

## Introduction

Inhibitory (GABAergic) interneurons of the cerebral cortex are critical for coordinating a wide range of cognitive and emotional functions. In the developing forebrain, cortical interneurons are born in the medial or caudal ganglionic eminence (MGE or CGE)^1–4^, or the preoptic area^5^, and migrate tangentially into the neocortex and hippocampus during late embryonic development. In the mature cerebral cortex^6,7^, a diverse population of GABAergic interneurons exists, each with distinct morphologies, physiologies and circuit functions. Loss (or dysfunction) of GABAergic neurons, particularly in hippocampus, has been linked to the development of epilepsy^8–12^, as well as intellectual disability, autism and psychiatric disorders^13–16^.

In hippocampus, the dentate gyrus subregion acts as a gatekeeper of information flow from cortex to hippocampus proper^17–19^. Granule cells are the main principal neuron of the dentate gyrus and receive strong local inhibition from GABAergic interneurons^6^. This robust inhibitory constraint, in concert with the unique anatomical and functional properties of the dentate gyrus, allows for only a small fraction of the total granule cell population to be activated simultaneously by excitatory input from the entorhinal cortex at any given time^20–23^. This ‘sparse population coding’ feature of dentate gyrus circuits is thought to be essential for memory formation and retrieval, and for pattern separation^20,21^. Robust local inhibition in the dentate gyrus may also protect against seizure genesis^17,19^.

GABAergic interneurons may be particularly susceptible to gene mutations associated with neuronal migration disorders due to their relatively long migratory routes to the cortex. Lissencephaly is a rare neuronal migration disorder often caused by mutations, or deletions, in platelet-activating factor acetyl hydrolase IB subunit alpha (Pafah1b1*;* also called *Lissecephaly-1* or *Lis1*)^24–26^. Pafah1b1 is an essential regulator of dynein-mediated motility, as well as mitosis, nuclear positioning, and microtubule organization^27^. Mice with heterozygous deletion of Pafah1b1 exhibit many phenotypes of human lissencephaly, including defects in neuronal migration, disorganization of cortical and hippocampal lamination, deficits in spatial learning and epilepsy^28–32^. In acute slice cultures generated from Pafah1b1^+/−^ mice, putative interneurons migrate ~25% more slowly away from the embryonic ganglionic eminence^33,34^, and excitatory synapses on dendrites of dissociated hippocampal interneurons are disorganized^35^. In postnatal mice, GABAergic interneurons are present in Pafah1b1^+/−^ hippocampus^30,36^, but lamination of individual cell types and inhibitory input to CA1 pyramidal neurons is disrupted in these animals^36,37^. However, it is not known whether slowly migrating interneurons eventually ‘catch-up’, arriving late in the cerebral cortex, or if a regional or subtype-specific change in interneuron number, density or synaptic inhibition exists in Pafah1b1^+/−^ mice.

In this study, we investigated the effect of Pafah1b1 haploinsufficiency on GABAergic inhibition in the dentate gyrus using Pafah1b1^+/−^ mice crossed to a GAD67-GFP reporter line. We show evidence of slowed GABA neuron migration into the hippocampus *in vivo*, and a subtype-specific reduction of interneurons and subsequent synaptic inhibition in the dentate gyrus. Finally, young-adult Pafah1b1^+/−^ mice develop spontaneous electrographic seizures and deficits in contextual memory.

## Results

### Interneuron migration and density are reduced in Pafah1b1^+/−^ dentate gyrus

**Figure 1:**
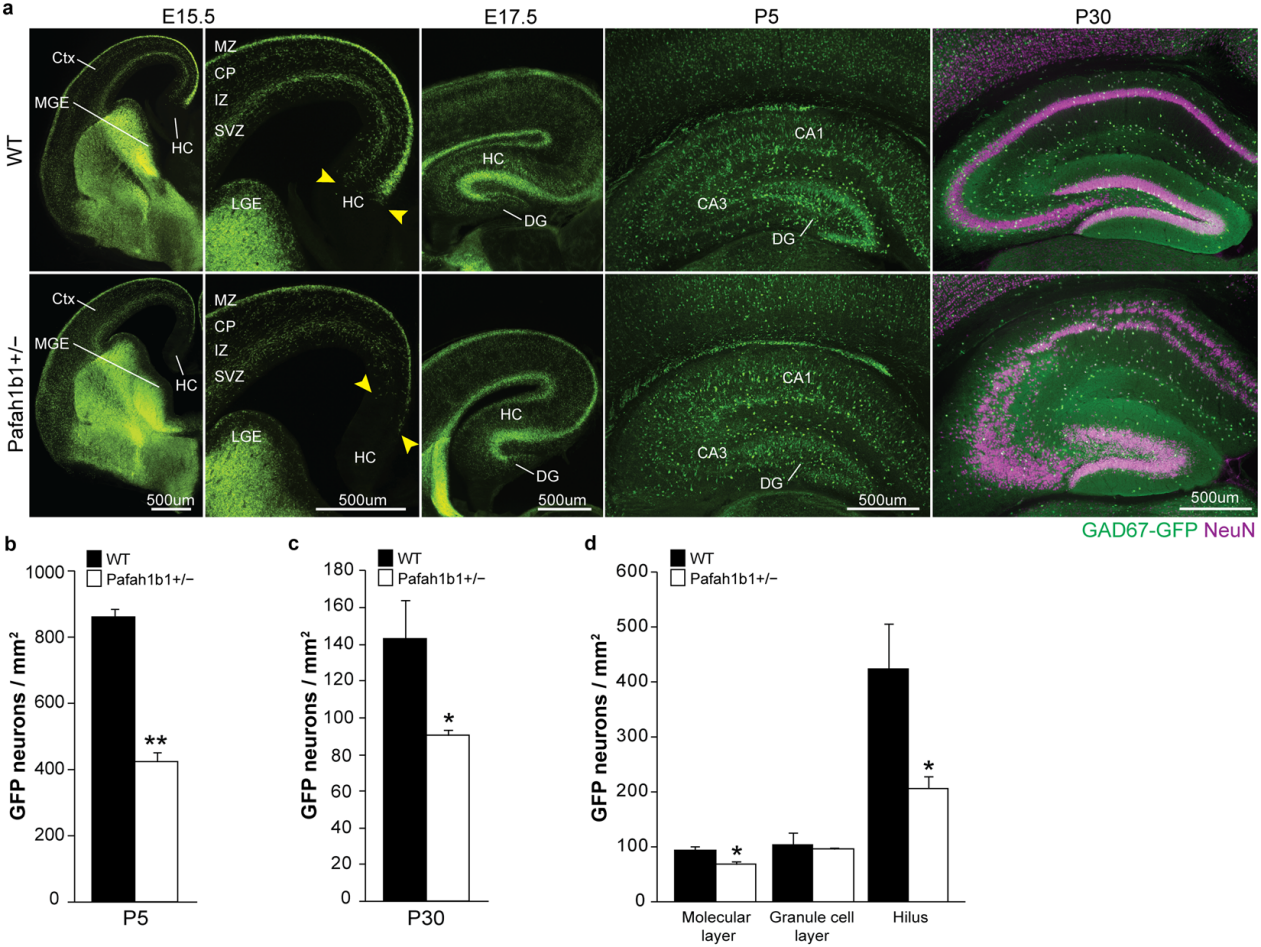
Density of GAD67-GFP cells is reduced in Pafah1b1^+/−^ dentate gyrus. (**a**) Distribution of GAD67-GFP+ interneurons (green) in wild-type and Pafah1b1^+/−^ mice at E15.5, E17.5, P5 and P30 and NeuN (magenta) at P30 (n = 4 for each genotype at each age). Yellow arrows indicate the leading GFP+ cells. Abbreviations: HC, hippocampus; DG, dentate gyrus; Ctx, cortex; MZ, mantle zone; CP, cortical plate; IZ, intermediate zone; SVZ, subventricular zone; MGE, medial ganglionic eminence; LGE, lateral ganglionic eminence. (**b**) Quantification of GFP+ neuron density in dentate gyrus at P5. (**c**) Quantification of GFP+ neuron density in dentate gyrus at P30. (**d**) Quantification of GFP+ neuron density within each sub-region of dentate gyrus at P30. Error bars represent s.e.m., *p < 0.05, **p < 0.01.

To visualize the effect of Pafah1b1^+/−^ on developing GABAergic interneurons *in vivo*, we crossed Pafah1b1^+/−^ mice^28^ with a GAD67-GFP knockin reporter line labeling nearly all GABAergic interneurons^38^ (Fig. 1a). We first examined the position of GFP-labeled neurons at E15.5, E17.5, P5 and P30. At E15.5, we found that GFP^+^ interneurons of wild-type mice had fully migrated into the developing hippocampus (n = 4 mice; Fig. 1a). However, we observed a delay in tangential migration of GAD67-GFP-labeled interneurons in Pafah1b1^+/−^ mice at E15.5 and an overt disorganization in the migratory routes of GAD67-GFP+ cells. GFP+ cells were absent from hippocampus in mutants at this age (n = 4 of 4 mice). By E17.5, GFP+ cells could be found throughout the hippocampus and dentate gyrus of both genotypes, indicating these cells eventually reached the hippocampal primordium in mutant animals. However, at P5, the density of GFP^+^ interneurons in dentate gyrus was reduced in Pafah1b1^+/−^ mice compared to wild-type littermates (wild-type: 861.4 ± 25.4 cells per mm^2^, n = 4; Pafah1b1^+/−^, 422.1 ± 31.7 cells per mm^2^, n = 4; p < 0.01, two-tailed t test) (Fig. 1b). Interneuron density remained significantly reduced in Pafah1b1 mutants at P30 (wild-type: 143.4 ± 20.9 cells per mm^2^, n = 4; Pafah1b1^+/−^, 90.4 ± 3.2 cells per mm^2^, n = 4; p < 0.05, two-tailed t test) (Fig. 1c); a time when principal cell layer dispersion was obvious in these animals (Fig. 1a). Next, we assessed whether there were any layer-specific loss or abnormal positioning of GABA neurons in dentate gyrus at P30. In both wild-type and Pafah1b1 mutants, GFP+ GABA neurons were primarily found in the molecular layer and hilus, and to a lesser extent, the granule cell layer of P30 animals. We detected a loss of GFP+ cell bodies positioned in the hilus (wild-type: 425.6 ± 82.2 cells per mm^2^, n = 4; Pafah1b1^+/−^, 205.8 ± 23.0 cells per mm^2^, n = 4; p < 0.05, two-tailed t test) and molecular layer (wild-type: 93.0 ± 8.1 cells per mm^2^, n = 4; Pafah1b1^+/−^, 68.5 ± 5.0 cells per mm^2^, n = 4; p < 0.05, two-tailed t test) (e.g., regions normally occupied by dense numbers of inhibitory neurons), with no change in the number of GFP + cells in the granule cell layer (wild-type: 103.2 ± 24.1 cells per mm^2^, n = 4; Pafah1b1^+/−^, 96.0 ± 2.9 cells per mm^2^, n = 4; p = 0.78, two-tailed t test) (Fig. 1d). Thus, tangential migration of GABAergic interneurons is slowed in Pafah1b1^+/−^ mice, consistent with findings from earlier *in vitro* studies, and there are fewer GABA neurons in the dentate gyrus of these animals.

**Figure 2:**
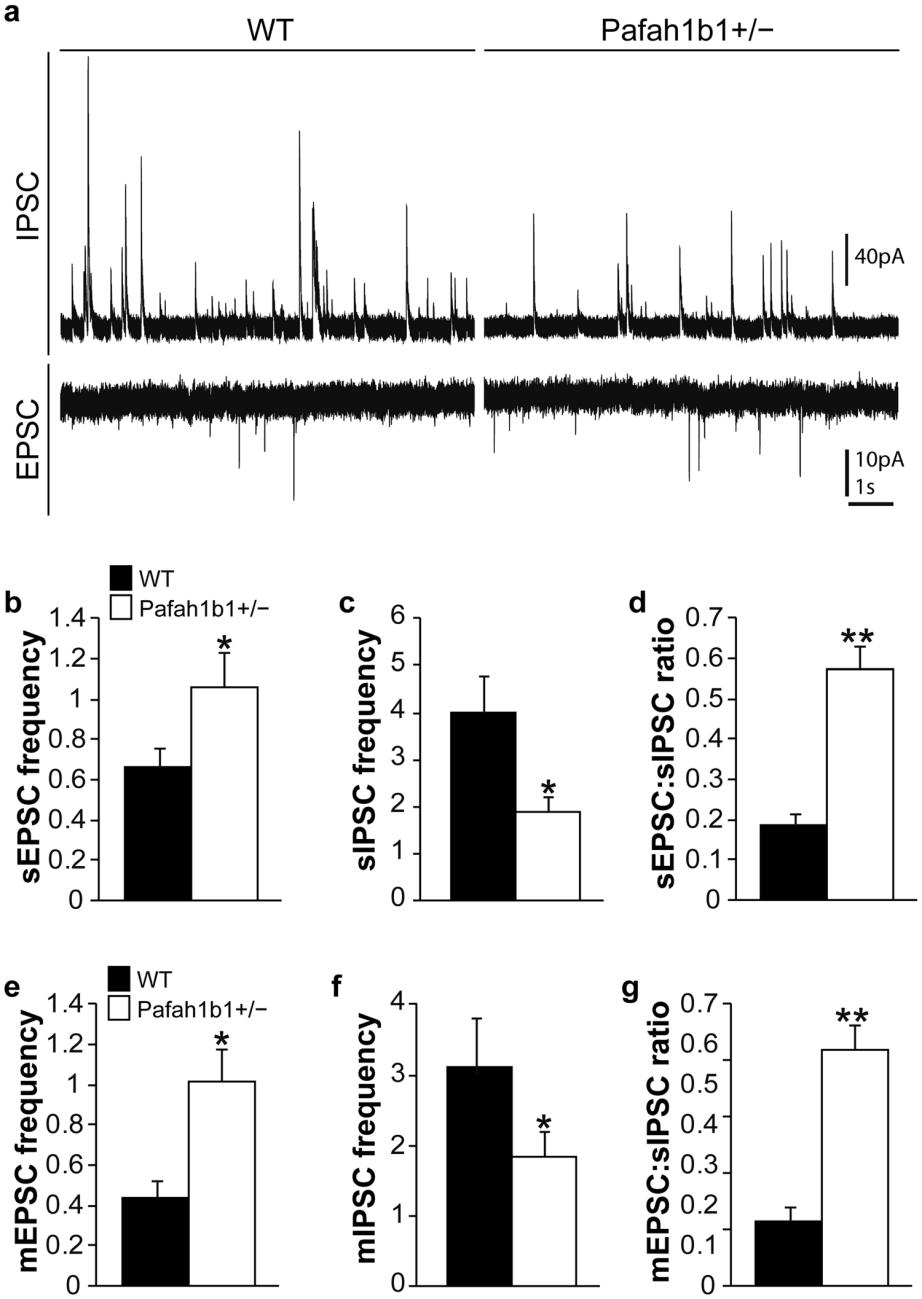
Fewer PV+ and SST+, but not CR+, GABAergic interneurons in Pafah1b1^+/−^ dentate gyrus. (**a**) Immunostaining for GAD67-GFP (green), PV (magenta) and DAPI (blue) in wild-type and Pafah1b1^+/−^ mice at P30. (**b**) Immunostaining for GAD67-GFP (green), SST (magenta) and DAPI (blue) in wild-type and Pafah1b1^+/−^ mice at P30. (**c**) Immunostaining for GAD67-GFP (green), CR (magenta) and DAPI (blue) in wild-type and Pafah1b1^+/−^ mice at P30. (**d**–**f**) Quantification of PV- (**d**), SST- (**e**) and CR-expressing (**f**) GABA neurons in dentate gyrus of wild-type and Pafah1b1^+/−^ mice at P30 (n = 4 for each genotype). Error bars represent s.e.m., *p < 0.05, **p < 0.01.

To examine whether individual GABAergic cell types were preferentially lost in Pafah1b1^+/−^ dentate gyrus, we performed a series of immunostaining experiments at P30. We found a 45% reduction of parvalbumin (PV) interneurons (wild-type: 68.5 ± 11.7 cells per mm^2^, n = 4; Pafah1b1^+/−^: 37.7 ± 8.9 cells per mm^2^, n = 5; p < 0.05, two-tailed t test) and a 56% reduction in somatostatin (SST) interneurons (wild-type: 84.1 ± 8.6 cells per mm^2^, n = 4; Pafah1b1^+/−^: 35.4 ± 3.9 cells per mm^2^, n = 4; p < 0.01, two-tailed t test) in dentate gyrus of Pafah1b1^+/−^ mice compared to wild-type littermates (Fig. 2a and b). In contrast, we did not find a change in the density of calretinin (CR) interneurons (wild-type: 23.5 ± 3.5 cells per mm^2^, n = 4; Pafah1b1^+/−^: 20.6 ± 4.1 cells per mm^2^, n = 5; p = 0.51, two-tailed t test) (Fig. 2c). Thus, there is a cell-type specific change in the density of interneurons in dentate gyrus of Pafah1b1^+/−^ mice.

### Synaptic input to dentate granule cells is altered in Pafah1b1^+/−^ mice

**Figure 3:**
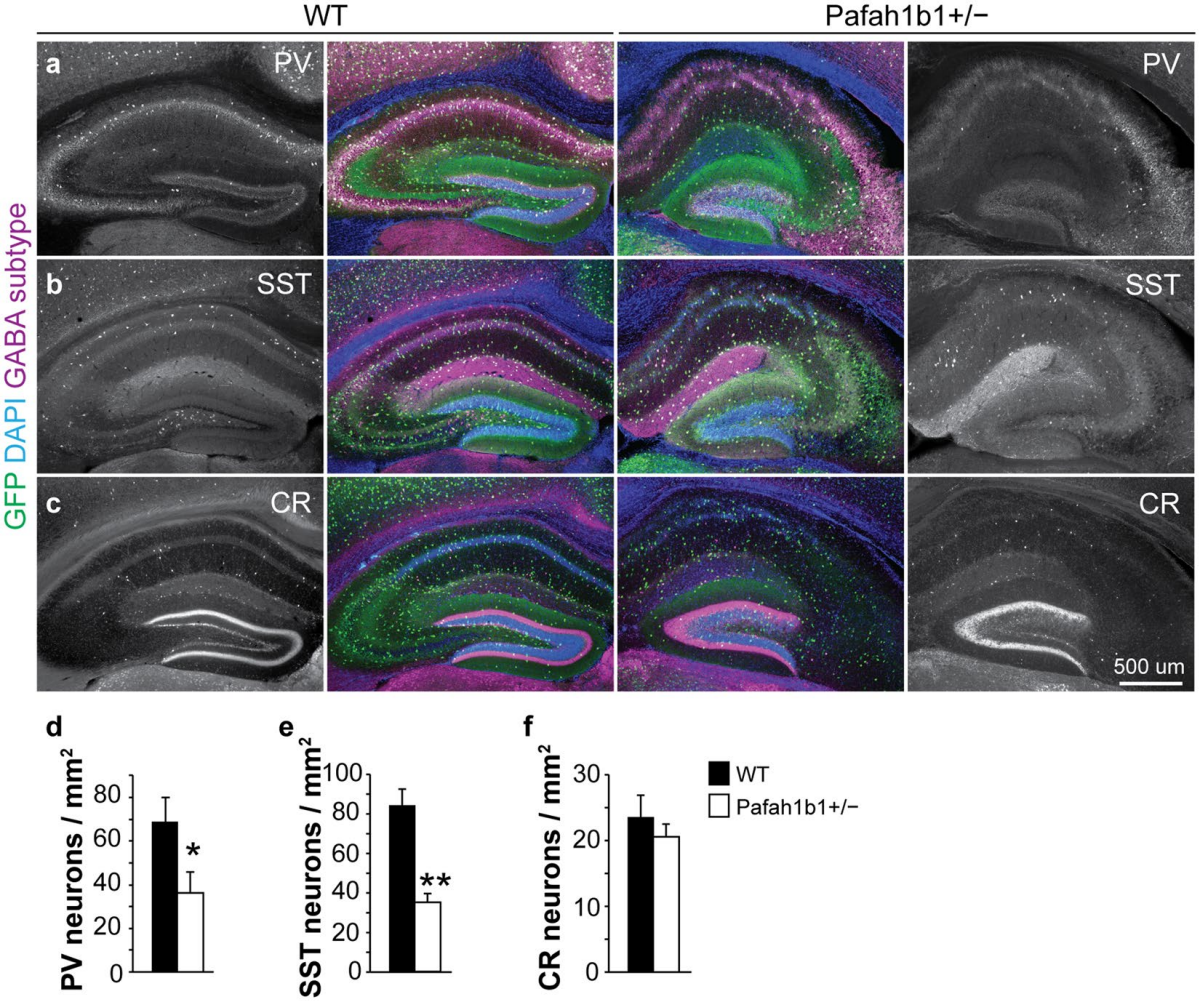
Dramatic change in synaptic inhibition and excitation in dentate granule cells of Pafah1b1^+/−^ mice. (**a**) Example voltage-clamp recordings of sIPSCs and sEPSCs recorded from granule cells in slices of a wild-type control and Pafah1b1 mutant. (**b–d**) Average sEPSC frequency (**b**), sIPSC frequency (**c**) and sEPSC:sIPSC ratio (**d**) in granule cells of wild-type and Pafah1b1^+/−^ mice. (**e**–**g**) Average mEPSC frequency (**e**), mIPSC frequency (**f**) and mEPSC:sIPSC ratio (**g**) in granule cells of wild-type and Pafah1b1^+/−^ mice. Error bars represent s.e.m., *p < 0.05, **p < 0.01.

Changes in the balance between excitation and inhibition underlies many forms of epilepsy, intellectual disability and autism spectrum disorder^9,10,12,13,15,16^. To evaluate excitation/inhibition (E/I) synaptic balance in the dentate gyrus of Pafah1b1^+/−^ mice, we obtained whole-cell voltage-clamp recordings from granule cells in acute hippocampal slices at P30–35 (Fig. 3). For these experiments, excitatory postsynaptic currents (EPSCs) were obtained at −70 mV and inhibitory postsynaptic currents (IPSCs) at 0 mV in the same cells (Fig. 3a). Mean spontaneous EPSC frequency was significantly higher in granule cells of Pafah1b1^+/−^ mice versus controls (wild-type: 0.66 ± 0.1 Hz, n = 9 cells from 4 animals; Pafah1b1^+/−^: 1.06 ± 0.16 Hz, n = 12 cells from 4 animals; p < 0.05, two-tailed t test). We did not detect a difference between treatment groups in mean EPSC amplitude (wild-type: 8.9 ± 0.88 pA; Pafah1b1^+/−^: 7.0 ± 0.3 pA; p = 0.1, two-tailed t test), 10–90% rise-time (wild-type: 1.19 ± 0.07 ms; Pafah1b1^+/−^: 1.15 ± 0.06 ms; p = 0.54, two-tailed t test) or decay time constant (wild-type: 4.3 ± 0.22 ms; Pafah1b1^+/−^: 4.0 ± 0.15 ms; p = 0.24, two-tailed t test). Similar to spontaneous events, miniature EPSC frequency recorded in the presence of 2 μM TTX was also significantly greater in Pafah1b1 mutants versus wild-type controls (wild-type: 0.45 ± 0.09 Hz, n = 9 cells; Pafah1b1^+/−^: 1.0 ± 0.3 Hz, n = 12 cells; p < 0.05, two-tailed t test). These findings are consistent with our previous reports showing greater excitatory input onto granule cells and CA1 pyramidal neurons in Pafah1b1^+/−^ mice, which is likely due to enhancement of pre-synaptic glutamate release in these animals^31,32^.

Next, we evaluated spontaneous and miniature IPSCs in the same cells used to measure EPSCs. Mean spontaneous IPSC frequency was significantly reduced in granule cells of Pafah1b1 mutants versus controls (wild-type: 4.0 ± 0.8 Hz, n = 9 cells; Pafah1b1^+/−^: 1.88 ± 0.34 Hz, n = 12 cells; p < 0.05, two-tailed t test). We did not detect a difference between treatment groups in mean IPSC amplitude (wild-type: 25.4 ± 4.2 pA; Pafah1b1^+/−^: 23.3 ± 4.8 pA; p = 0.71, two-tailed t test), 10–90% rise-time (wild-type: 1.5 ± 0.16 ms; Pafah1b1^+/−^: 1.4 ± 0.2 ms; p = 0.78, two-tailed t test) or decay time constant (wild-type: 12.9 ± 1.6 ms; Pafah1b1^+/−^: 12.2 ± 0.89 ms; p = 0.71, two-tailed t test). Similar to spontaneous events, miniature IPSC frequency recorded in the presence of 2 μM TTX was also significantly lower in Pafah1b1 mutants versus wild-type controls (wild-type: 3.1 ± 0.8 Hz, n = 9 cells; Pafah1b1^+/−^: 1.85 ± 0.3 Hz, n = 12 cells; p < 0.05, two-tailed t test). Finally, E/I balance was analyzed by calculating the ratio of the frequency of EPSCs to IPSCs. We found a nearly four-fold significant increase in the E to I ratio for both miniature and spontaneous events in granule cells of Pafah1b1^+/−^ mice (Fig. 3d,g). Taken together, there is a substantial shift in the balance between synaptic excitation and inhibition in the dentate gyrus of Pafah1b1^+/−^ mice.

### Pafah1b1^+/−^ mice have long-term deficits in context-based memory

**Figure 4:**
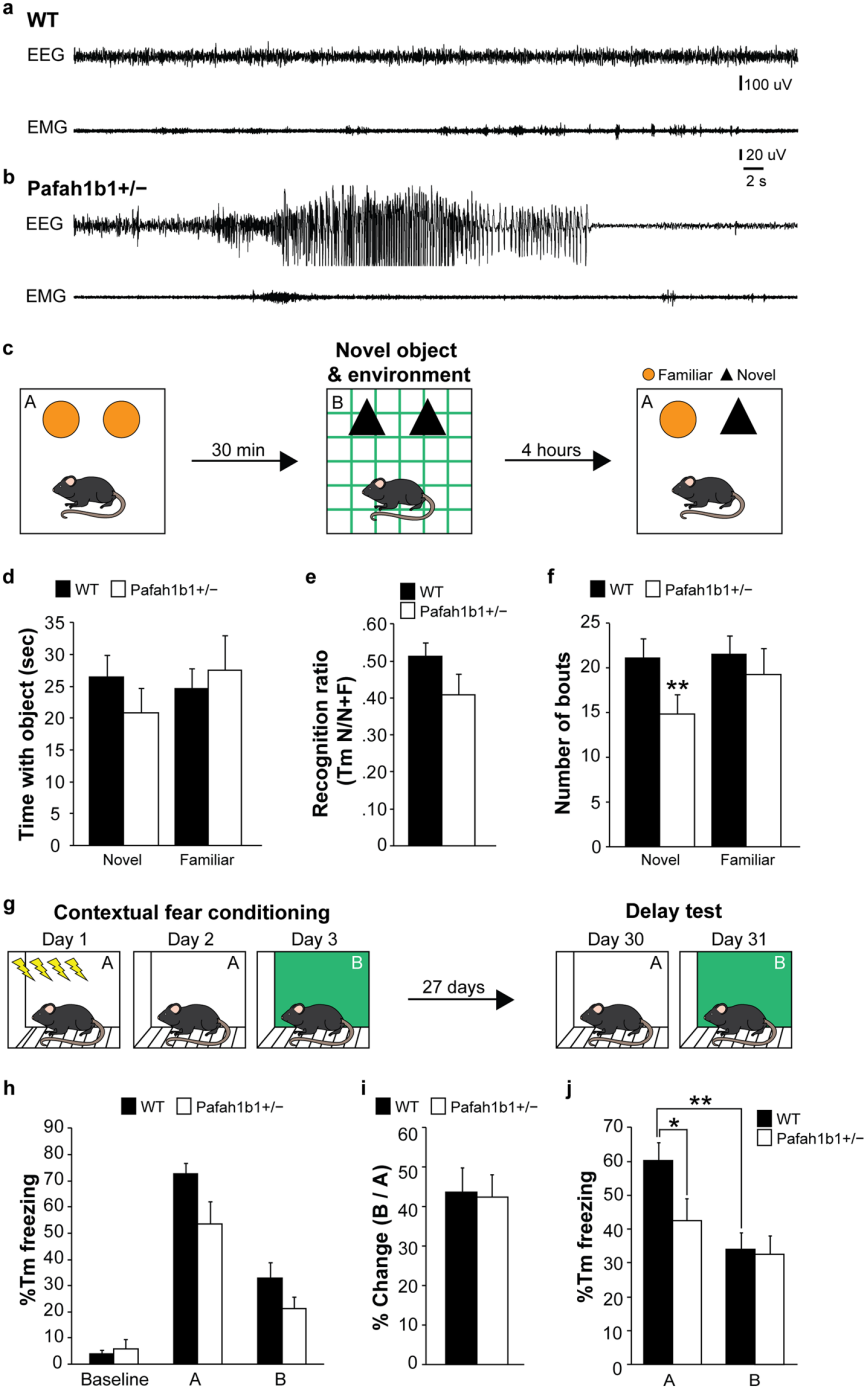
Pafah1b1^+/−^ mice display seizures and deficits in context memory. (**a**) Example EEG and corresponding EMG recording from a wild-type mouse. (**b**) Example of a spontaneous seizure recorded in a Pafah1b1 mutant. (**c**) Object-based pattern separation protocol (n = 12 wild-type and n = 10 Pafah1b1 mutants). (**d**) Mean time spent with each object during the test trial (trial 3). (**e**) Mean recognition ratio (Tm N/N + F) during the test trial (trial 3). (**f**) Mean number of bouts made for each object during the test trial (trial 3). (**g**) Contextual fear conditioning protocol (n = 12 wild-type and n = 10 Pafah1b1 mutants). (**h**) Percent time freezing before conditioning (baseline) and in context A and B during the two days following conditioning. (**i**) Ratio of percentage of time freezing in context B versus context A. (**j**) Percent time freezing during the delay test on days 30 and 31 of testing. Error bars represent s.e.m., *p < 0.05, **p < 0.01.

Pafah1b1 mutant mice develop handling-induced seizures, spontaneous epilepsy and early death^28,31^. To further examine whether the mutant mice used in our study, which were crossed into a mixed C57BL/6J and CD1 background strain to visualize GAD67-GFP cells, also displayed spontaneous electrographic seizures, we performed continuous EEG monitoring (7 d, 24 h/d) from freely behaving animals at 6–8 weeks of age (Fig. 4a,b). In two of the four mutant mice examined, we observed spontaneous seizures consisting of high-frequency, high-voltage, rhythmic activity with clear onset and termination of ictal events (frequency: 0.797 and 0.31 seizures per day, duration 34.2 ± 3.0 and 38.4 ± 3.1 s). Electrographic seizures or high-voltage spiking were never observed in wild-type animals (n = 5).

Hippocampal GABA neurons play an active role in contextual learning^39^, and mice with selective GABA neuron loss in the hippocampus can develop deficits in learning and memory^40,41^. To evaluate context discrimination and learning in Pafah1b1^+/−^ mice, we performed two behavioral assays. First, we evaluated 2 month old Pafah1b1 mutants and wild-type littermates in an object-based pattern separation assay (Fig. 4c). This task is a modified version of the object recognition task, and it assesses the cognitive aspect of pattern separation in the absence of an emotional component^42^. Animals were exposed to two trials, spaced 30 minutes apart, and allowed to freely interact with two sets of identical objects within an open field chamber for 10 minutes per trial. Each environment was distinct in the pattern of the chamber (plain white versus grid pattern), the type of object in the chamber (plastic blocks versus glass flasks) and odor (1% acetic acid versus 30% ethanol). Four hours later, mice were tested in a third trial to evaluate interactions with each object. We did not detect a difference between wild-type and Pafah1b1 mutants in time spent exploring either the novel object (wild-type: 26.5 ± 3.5 sec, n = 12; Pafah1b1^+/−^: 20.8 ± 3.9 sec, n = 10; p = 0.3, two-tailed t test) or the familiar object (wild-type: 24.6 ± 3.3 sec, n = 12; Pafah1b1^+/−^: 27.5 ± 5.6 sec, n = 10; p = 0.65, two-tailed t test) (Fig. 4d). We also calculated a recognition ratio as the time spent exploring the novel/total time spent exploring the novel and familiar object; we did not find a difference between groups (wild-type = 0.51 ± 0.037; Pafah1b1^+/−^ = 0.41 ± 0.056; p = 0.13, two-tailed t test) (Fig. 4e). Finally, we analyzed the number of bouts of interactions each mouse made with an object. We found that Pafah1b1^+/−^ mice had significantly fewer bouts with the novel object compared to controls (wild-type = 21.2 ± 2.17; Pafah1b1^+/−^ = 14.9 ± 2.4; p < 0.01, two-tailed t test); no difference in the number of bouts made with familiar object was detected (wild-type = 21.5 ± 2.1; Pafah1b1^+/−^ = 19.0 ± 3.1; p = 0.5, two-tailed t test) (Fig. 4f).

Second, we examined contextual fear conditioning in 2 month old Pafah1b1^+/−^ mice and wild-type littermates (Fig. 4g). As expected, we observed very little freezing activity of any wild-type or mutant animal before the initial testing phase (Fig. 4h). Following fear conditioning, mice of both genotypes displayed a significantly greater percentage of time freezing when placed in context A, the chamber in which they received the foot shocks, versus context B, the chamber in which no foot shocks were applied. Pafah1b1^+/−^ mice expressed similar levels of contextual fear conditioning as wild-type mice (Fig. 4h). To determine the degree of conditioning between groups, we calculated the percentage of time freezing in context B versus time freezing in context A. Animals had approximately 40% change in freezing time between the two chambers, and we did not detect a significant difference between wild-type and Pafah1b1^+/−^ mice (wild-type = 0.44 ± 0.06, n = 12; Pafah1b1^+/−^ = 0.43 ± 0.06, n = 10; p = 0.9, two-tailed t test) (Fig. 4i). Animals were then returned to their home cages and re-tested 27 days later in a delay test. Wild-type mice displayed a significantly greater percentage of time freezing when placed in context A, the chamber in which they received the foot shocks, versus context B (context A = 60.4 ± 5.2 %Tm freezing; context B = 33.9 ± 4.97 %Tm freezing; p < 0.01, two-tailed t test); and the percentage of time spent freezing was comparable to the initial testing performed 27 days earlier. However, percentage of time freezing in context A was significantly reduced in Pafah1b1^+/−^ mice compared to wild-type littermates (wild-type = 60.4 ± 5.2 %Tm freezing; Pafah1b1^+/−^ = 42.5 ± 6.6 %Tm freezing; p < 0.05, two-tailed t test). No difference was detected in the percentage of time freezing in chamber A versus chamber B for Pafah1b1^+/−^ mice (context A = 42.5 ± 6.6 %Tm freezing; context B = 32.5 ± 5.7 %Tm freezing; p = 0.3, two-tailed t test). Taken together, Pafah1b1^+/−^ mice develop spontaneous seizures and deficits in remote contextual memory but short-term contextual learning appears relatively intact.

## Discussion

Pafah1b1^+/-^ mice develop a severe neuronal migration disorder, including abnormal positioning of glutamatergic principal neurons and GABAergic interneurons^30,32,37,43,44^. In this study, we found incorporation of GABAergic interneurons into dentate gyrus is disrupted by Pafah1b1 haploinsufficiency. Novel findings reported here include i) slowed tangential migration of young GAD67-GFP + interneurons into the embryonic hippocampus of Pafah1b1^+/−^ mice, ii) decreased density of GABA neurons in the malformed dentate gyrus once development was complete (including fewer PV and SST subtypes in Pafah1b1^+/−^ mice), iii) a dramatic shift in synaptic E/I balance in dentate granule cells, due in part to a substantial loss of synaptic inhibition and iv) long-term deficits in context-based memory. We also reproduce previously reported phenotypes of these animals: i) aberrant interneuron migration - first reported by McMannus *et al*., 2004 showing reduced migratory speed away from embryonic ventral telencephalon in slice cultures and reproduced in the current study showing less advanced positioning of GAD67-GFP cells in brain sections of Pafah1b1 mutants, ii) spontaneous electrographic seizures - first reported by Greenwood *et al*., 2009 and reproduced on a different genetic background strain in the current study and iii) increases in EPSC frequency onto dentate granule cells - first reported by Hunt *et al*., 2012 and replicated in the current study.

Our observations of slowed interneuron migration is consistent with earlier studies reporting 20–30% reduction in interneuron migration speed away from the ganglionic eminence in Pafah1b1^+/−^ embryonic slice cultures^33,34^. In human tissue studies, 19–20 week old fetuses with Miller-Dieker syndrome had a ten-fold significant reduction of CR-expressing interneurons in neocortex, but deficits in CR interneuron number, while still reduced, were not significantly different from control samples^45^. These types of findings raise two intriguing possibilities: i) slowed migration of GABA neurons from the ganglionic eminences might be due to developmental delay in Pafah1b1^+/−^ mice, and GABA neurons are simply born at a later date in these animals; or ii) perhaps GABA neurons ‘catch up’ later on in development and eventually populate the cortex and hippocampus in appropriate numbers. Previous work found dislamination, but not a loss, of individual interneuron subtypes in hippocampal area CA1 of Pafah1b1^+/−^ mice^37^. However, Pafah1b1^+/−^ interneurons have not previously been tracked from embryonic ages into adulthood, and dentate gyrus is one of the most distant cortical regions to be populated by GABAergic interneurons^46^. Our findings indicate GABAergic neurons do not simply ‘catch up’ in Pafah1b1^+/−^ mice, because deficits in interneuron density persist into maturity, at least in the dentate gyrus. However, we cannot rule out a potential contribution of increased interneuron death in Pafah1b1 mutants, either occurring naturally during development or as a result of spontaneous seizures. Interestingly, mice overexpressing Pafah1b1 have a similar delay in interneuron tangential migration^47^, suggesting GABAergic inhibition may be similarly disrupted in patients with Pafah1b1 duplications. Nevertheless, our findings support the idea that Pafahb1b haploinsufficieny is associated with brain region- and subtype-specific reductions in GABAergic interneurons.

Reduced synaptic inhibition in dentate gyrus of Pafah1b1 mutants is consistent with our observation of fewer GABAergic interneurons in this region. Other studies have shown anatomical disorganization of glutamatergic synapses on hippocampal interneurons. We cannot rule out the possibility that functional changes in interneuron excitability also contributes to the overall reduced network-driven inhibition observed in Pafah1b1^+/−^ dentate gyrus. However, excitatory drive onto hippocampal interneurons is increased, not decreased, in Pafah1b1^+/−^ mice^36^. We also detected a reduction in frequency of miniature IPSCs in mutants, which is consistent with a loss of inhibitory synapses onto dentate granule cells.

Pafah1b1 mutants have occasional spontaneous seizures as well as subtle impairments in motor performance, spatial learning and social interaction^29,31,48^. Here, we extend these prior behavior studies by reporting deficits in context-based memory in Pafah1b1^+/−^ mice. Considering the extensive neuronal and synaptic disorganization in these animals, it is surprising that seizures and/or behavioral problems are not more robust. Dentate granule cells are normally under strong inhibitory control, which is thought to be critical for the specialized functions of dentate gyrus^19,23,49^. In addition to deficits in synaptic inhibition, Pafah1b1 haploinsufficiency produces a substantial enhancement of pre-synaptic glutamate transmission in hippocampus and accelerated integration of adult-born granule cells in dentate gyrus^31,32,43,44^. Other systems disrupted by Pafah1b1^+/−^ (e.g., cytoskeleton) or non-neuronal cell types implicated in epilepsy (e.g., astrocytes) are also likely involved. It will be important to investigate in future studies whether functional changes in Pafah1b1^+/−^ circuits compensate for the dramatic structural abnormalities apparent in these animals, perhaps by dissociating Pafah1b1^+/−^ mutation from the underlying malformation^32^.

Uncontrolled epilepsy and severe intellectual disability are serious problems for individuals with lissencephaly. Our findings that GABA neuron migration and function is altered by Pafah1b1 haploinsufficiency is critically important to our understanding of malformation-associated epilepsies and for identifying new therapies.

## Methods

### Animals

All experiments were first approved by the University of California, San Francisco Animal Care and Use Committee and adhered to National Institutes of Health guidelines and regulations for the Care and Use of Laboratory Animals. Pafah1b1^+/−^ male mice^28,32^ were mated with female hemizygous glutamic acid decarboxylase - enhanced green fluorescence protein (GAD67-GFP) knock-in mice^38^ to produce Pafah1b1^+/−^; GAD67-GFP mice. All animals were bred in house and maintained on a background strain that was a mixture of C57BL/6 J and CD1 or a C57BL/6J background (context behavior assays). Experiments were performed on male and female littermates between E15.5 and P75. Embryos were obtained from two litters for experiments at E15.5 and E17.5. Sex of animals >P30 was comparable among the genotypes: 18 wild-type and 18 Pafah1b1^+/−^ male mice and 11 wild-type and 8 Pafah1b1^+/−^ female mice (Χ^2^ = 0.311, p = 0.58). Sex of mice ≤ P5 was not recorded. No significant gender differences were observed for any of the parameters analyzed in the study.

### Immunocytochemistry

Free-floating vibratome sections (50 μm) were processed according to our published protocol^32,50^. Primary antibody dilutions were as follows: chicken anti-GFP (1:500; Aves); mouse anti-PV (1:500; Sigma); rabbit anti-SST (1:200; Santa Cruz); rabbit anti-CR (1:1000; Millipore). Secondary antibodies included Alexa Fluor 488 and Alexa Fluor 594 (1:500; Life Technologies).

### Cell quantification

Fluorescently labeled sections (50 µm) were imaged using a Nikon Eclipse Ni-E microscope with an x20 objective and cell counts were performed using ImageJ, as described previously^32,50^. All cells that expressed GFP (or subtype maker) were counted in every sixth coronal section in all layers of the dentate gyrus (that is, 300 μm apart). To define the border of hilus/CA3, straight lines were drawn from the ends of the granule cell to the proximal end of the CA3 pyramidal cell layer. Four to six sections of the dorsal dentate gyrus were analyzed per animal and the values averaged to obtain a mean cell density (cells/mm^2^).

### Electrophysiology

Slice preparation and whole-cell patch-clamp recordings were performed according to our published protocols^12,32^. Coronal brain slices (300 μm) were prepared from P30–35 WT and Pafah1b1^+/−^ mice. ACSF (32–34 °C) contained (in mM) 124 NaCl, 3 KCl, 1.25 NaH_2_PO_4_-H_2_O, 2 MgSO_4_-7H_2_O, 26 NaHCO_3_, 10 dextrose, and 2 CaCl_2_ (pH 7.2–7.4, 300–305 mOsm/kg). Patch pipettes (2–4 MΩ) were filled with an internal solution, containing (in mM) 140 Cs^+^ gluconate, 1 NaCl, 5 EGTA, 10 HEPES, 1 MgCl_2_, 1 CaCl_2_, 3 KOH, 2 ATP, and 0.2% biocytin, pH 7.21. Spontaneous (s) and miniature (m) synaptic events were examined at a holding potential of −70 mV (EPSCs) or 0 mV (IPSCs). Tetrodotoxin (TTX, 2 μM) was added to the ACSF to isolate mIPSCs. Series resistance was typically <15 MΩ and was monitored throughout the recordings.

### EEG

EEG recordings were obtained using a time-locked video EEG monitoring system (Pinnacle Technologies) as previously described^31,50,51^. Mice were surgically implanted in the left and right frontoparietal cortex with electrodes. Each mouse was anesthetized with ketamine and xylazine (10 mg/kg and 1 mg/kg, i.p.) so that there was no limb-withdrawal response to a noxious foot pinch. Sterile, stainless-steel bone screw recording electrodes were placed epidurally through burr holes in the skull (one electrode on either side of the sagittal suture, approximately halfway between bregman and lambdoid sutures and ≈1 mm from the midline) using surface head-mount EEG hardware (Pinnacle Technologies). Electrodes were cemented in place with a fast-acting adhesive and dental acrylic. Two wires were laid on the shoulder muscles for EMG recording. Animals were allowed to recover for 3 d before experiments were initiated. Electrographic seizures were defined as high-frequency, high-voltage synchronized polyspike or paroxysmal sharp waves with amplitude >2-fold background that lasted ≥15 s. Experimental animals were monitored 24 h/d for a total of 7 continuous days. Electrographic EEG seizures were analyzed in control (n = 5) and mutant mice (n = 4) by an experimenter blinded to mouse genotype using SireniaScore software (Pinnacle Technologies) and confirmed by offline review.

### Object-based pattern separation

The object pattern separation task is a modified version of the novel object recognition task^42^. In this task, we used a 16in × 16in × 8.75in square open field arena made of smooth opaque white plastic. Two identical objects were used in two distinct arena environments per trial. Chamber A contained a plain white chamber, two identical plastic blocks and was cleaned with 1% acetic acid between trials. Animals were unable to move these objects. Chamber B contained a grid pattern created by striping the box with green laboratory tape, two identical glass laboratory flasks and was cleaned with 30% ethanol between trials. For this assay, we used 12 wild-type mice and 10 Pafah1b1^+/−^ littermates maintained on a C57BL/6J background. Mice were moved into the testing room, randomly distributed into two groups (experimenter was blind to animal genotype) and allowed to habituate to light and room for one hour prior to each phase of the experiment. One group was tested first in chamber A, then chamber B; the other group was tested first in chamber B, then chamber A. The task consisted of two acquisition trials of free exploration with a 30 minute time interval in-between, followed by a test trial 4 hours after the second session. In the first trial, two identical objects were placed symmetrically on a horizontal line in the arena, approximately 5 cm from the wall. Mice were placed in the front of the arena facing the wall and allowed to explore the objects for 10 minutes, after which they were returned to their home cage for 30 minutes. In the second trial (30 minutes after the first trial), mice were placed into the chamber not tested in the first trial. The mice were again allowed to explore this new chamber for 10 minutes, after which they were returned to their home cage for four hours. In the third trial, four hours after the second trial, mice were placed back into chamber A and allowed to explore the objects for 10 minutes. In this third trial, chamber A contained one object from chamber A and one object from chamber B. Over the three trials, the objects orientation and location in the chamber was always kept the same. Each session was videotaped from above the chamber using a camera on a tripod, and videos were analyzed offline using CleverSys TopScan software to measure the time spent with each object and bouts of interactions made with the object.

### Contextual Fear Conditioning

We performed contextual fear conditioning experiments using 12 wild-type mice and 10 Pafah1b1^+/−^ littermates maintained on a C57BL/6 J background. Mice were moved into the testing room, randomly distributed into five groups (experimenter was blind to animal genotype) and allowed to habituate to light and room for one hour prior to each phase of the experiment. On the first day (training), mice were placed in the fear conditioning apparatus (context A) and monitored for 5 min to measure baseline freezing activity. Then, four 2-second, 0.45 mA foot shocks were presented, separated by 120-second inter-trial intervals during which freezing was monitored, with a 60 second end period following the last foot shock. The next day (context test), mice were placed in the identical fear conditioning chamber for 8 minutes; no shock was presented and the amount of freezing was measured. On the third day (generalization test), mice were placed in a different fear conditioning chamber (context B; altered by inserting a black triangular insert, turning the fan off, spraying trays with acetic acid instead of Simple Green, and inserting a staggered-bar floor grid) and monitored for 8 minutes. At the conclusion of the three day testing period, mice were returned to the animal housing room. A delay test was performed 27 days later by measuring freezing behavior in context A, followed 24 hrs later by context B.

### Analyses and statistics

Data analysis was performed using pClamp 10.2 (Clampfit; Molecular Devices), MiniAnalysis 6.0 (Synaptosoft), Microsoft Excel, ImageJ and Systat 12 programs. A 2–5 min sample recording per cell was used for measuring EPSC or IPSC frequency, amplitude, 10–90% rise time, and decay time constant as previously described (Hunt *et al*., 2011; Hunt *et al*. 2012). For cell quantifications, cells were counted in sections from the dorsal half of the hippocampus of each animal. Experimental groups were compared by two-tailed Student’s t test or by one-way ANOVA, followed by a Tukey’s post hoc test. Data are expressed as mean ± SEM, and significance was set at p < 0.05.

### Data availability

All data generated or analyzed during this study are included in this published article.
