# Publisher Correction: PAFAH1B1 haploinsufficiency disrupts GABA neurons and synaptic E/I balance in the dentate gyrus

**DOI:** 10.1038/s41598-018-24610-w

**Published:** 2018-04-25

**Authors:** Matthew T. Dinday, Kelly M. Girskis, Sunyoung Lee, Scott C. Baraban, Robert F. Hunt

**Affiliations:** 1 Epilepsy Research Laboratory, Department of Neurological Surgery, University of California San Francisco, San Francisco, USA; 2 Department of Anatomy & Neurobiology, University of California Irvine, California, USA

Correction to: *Scientific Reports* 10.1038/s41598-017-08809-x, published online 15 August 2017

This Article contains an error in the order of the Figures. Figures 2 and 3 were published as Figures 3, and 2 respectively. The correct Figures 2 and 3 appear below as Figures 1 and 2 respectively. The Figure legends are correct.

**Figure 1:**
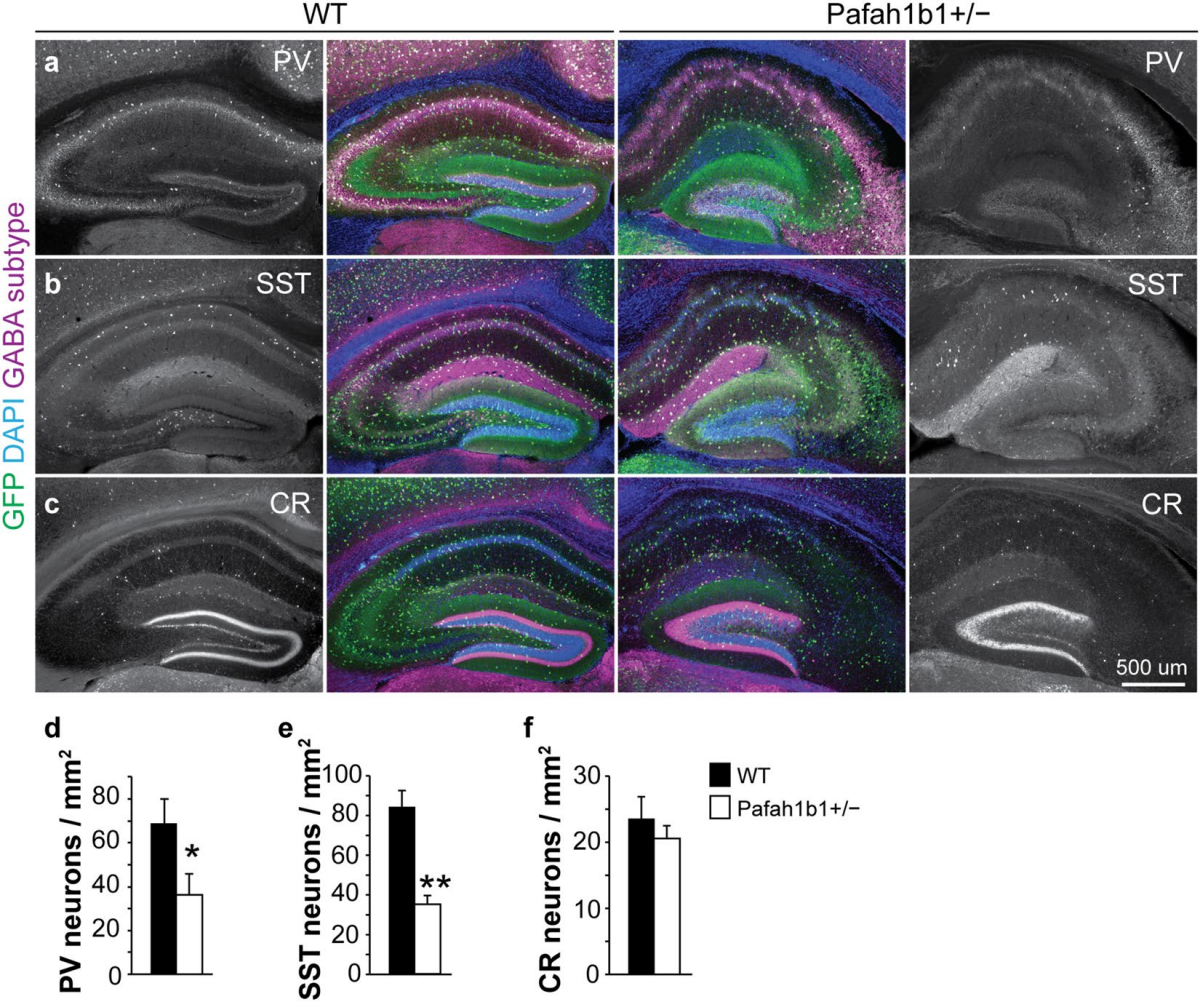
Fewer PV+ and SST+, but not CR+, GABAergic interneurons in Pafah1b1+/− dentate gyrus. (**a**) Immunostaining for GAD67-GFP (green), PV (magenta) and DAPI (blue) in wild-type and Pafah1b1+/− mice at P30. (**b**) Immunostaining for GAD67-GFP (green), SST (magenta) and DAPI (blue) in wild-type and Pafah1b1+/− mice at P30. (**c**) Immunostaining for GAD67-GFP (green), CR (magenta) and DAPI (blue) in wild-type and Pafah1b1+/− mice at P30. (d–f) Quantification of PV- (**d**), SST- (**e**) and CR-expressing (f) GABA neurons in dentate gyrus of wild-type and Pafah1b1+/− mice at P30 (n = 4 for each genotype). Error bars represent s.e.m., *p < 0.05, **p < 0.01.

**Figure 2:**
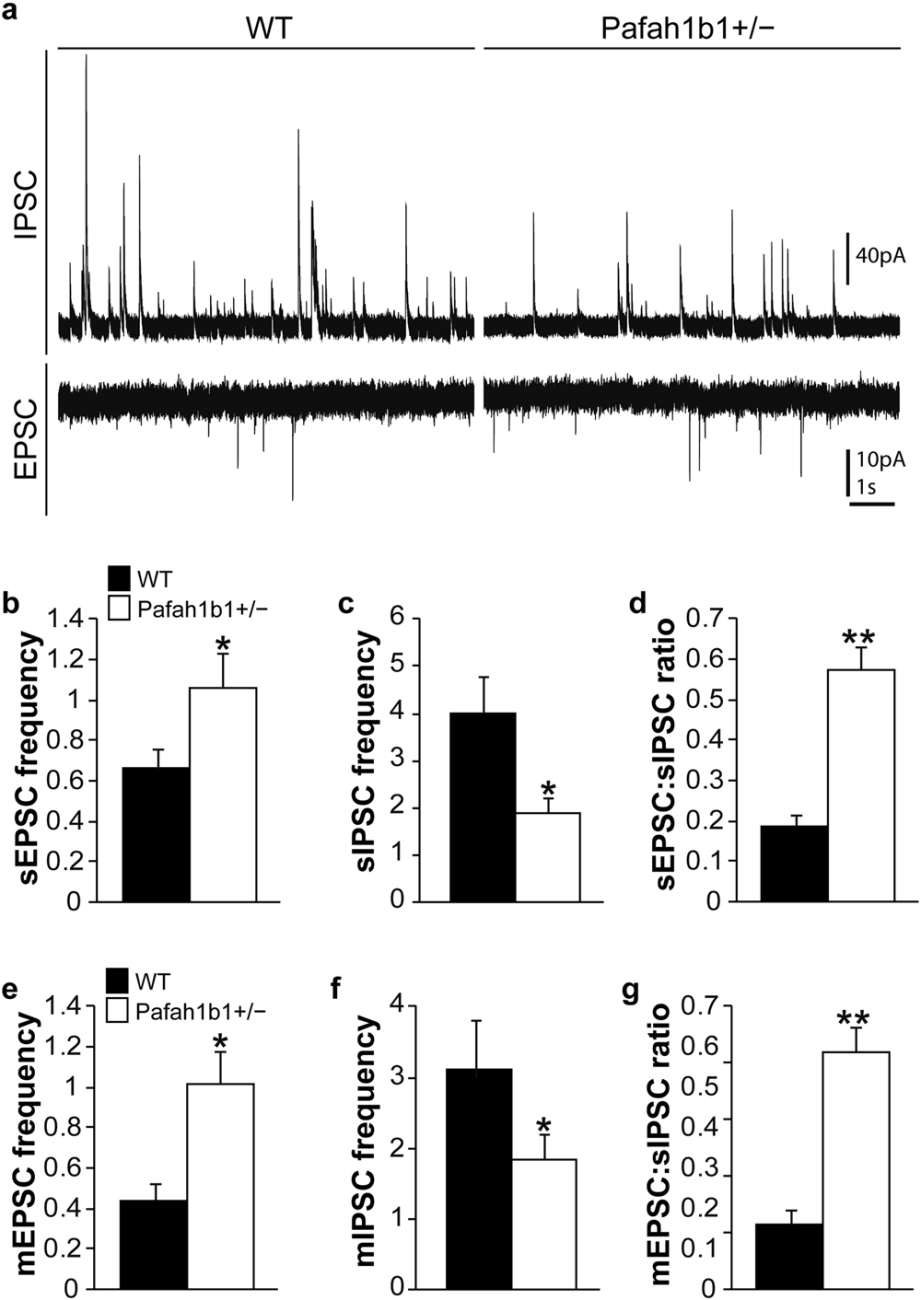
Dramatic change in synaptic inhibition and excitation in dentate granule cells of Pafah1b1+/− mice. (**a**) Example voltage-clamp recordings of sIPSCs and sEPSCs recorded from granule cells in slices of a wild-type control and Pafah1b1 mutant. (**b**–**d**) Average sEPSC frequency (**b**), sIPSC frequency (**c**) and sEPSC:sIPSC ratio (**d**) in granule cells of wild-type and Pafah1b1+/− mice. (**e**–**g**) Average mEPSC frequency (**e**), mIPSC frequency (**f**) and mEPSC:sIPSC ratio (**g**) in granule cells of wild-type and Pafah1b1+/− mice. Error bars represent s.e.m., *p < 0.05, **p < 0.01.

